# Retraction: Jiang, H.; Mei, Y.-F. SARS-CoV-2 Spike Impairs DNA Damage Repair and Inhibits V(D)J Recombination In Vitro. *Viruses* 2021, *13*, 2056

**DOI:** 10.3390/v14051011

**Published:** 2022-05-10

**Authors:** Hui Jiang, Ya-Fang Mei

**Affiliations:** 1 Department of Molecular Biosciences, The Wenner-Gren Institute, Stockholm University, SE-10691 Stockholm, Sweden; 2 Department of Clinical Microbiology, Virology, Umeå University, SE-90185 Umeå, Sweden

The published article [1] has been retracted. Following publication, the first author contacted the editorial office regarding an improper experimental design with the potential to significantly affect the integrity of the resultant experimental data. 

Adhering to our complaint procedure, an investigation was conducted. Both the chosen construct of the spike plasmid that contained a C-terminal fused with 6xHis tag and use of a GFP reporter system under overexpression conditions in the protocol were identified as having the potential to introduce significant ambiguity regarding the nature of the reported observations. The reliability of the results and conclusions presented have therefore been undermined. Furthermore, statements regarding the effect of the spike protein on the adaptive immunity are misleading as in this article no experiments related to the adaptive immunity were performed, and the full-length spike-based vaccine was not studied. Therefore, conclusions related to vaccine safety are not validated and lacked experimental support. This article [1] is retracted and shall be marked accordingly. This retraction was approved by the Editor-in-Chief of the journal *Viruses*.
